# Silicon-Based Microfabrication of Free-Floating Neural Probes and Insertion Tool for Chronic Applications

**DOI:** 10.3390/mi9030131

**Published:** 2018-03-16

**Authors:** Andreas Schander, Heiko Stemmann, Andreas K. Kreiter, Walter Lang

**Affiliations:** 1 Institute for Microsensors, -actuators and -systems (IMSAS), University of Bremen, Bremen 28359, Germany; wlang@imsas.uni-bremen.de; 2 Brain Research Institute, University of Bremen, Bremen 28359, Germany; stemmann@brain.uni-bremen.de (H.S.); kreiter@brain.uni-bremen.de (A.K.K.)

**Keywords:** neural probe, floating implantation, chronic recording, long-term stability, intracortical, PEDOT:PSS, polyimide, silicon probe

## Abstract

Bidirectional neural interfaces for multi-channel, high-density recording and electrical stimulation of neural activity in the central nervous system are fundamental tools for neuroscience and medical applications. Especially for clinical use, these electrical interfaces must be stable over several years, which is still a major challenge due to the foreign body response of neural tissue. A feasible solution to reduce this inflammatory response is to enable a free-floating implantation of high-density, silicon-based neural probes to avoid mechanical coupling between the skull and the cortex during brain micromotion. This paper presents our latest development of a reproducible microfabrication process, which allows a monolithic integration of a highly-flexible, polyimide-based cable with a silicon-stiffened neural probe at a high resolution of 1 µm. For a precise and complete insertion of the free-floating probes into the cortex, a new silicon-based, vacuum-actuated insertion tool is presented, which can be attached to commercially available electrode drives. To reduce the electrode impedance and enable safe and stable microstimulation an additional coating with the electrical conductive polymer PEDOT:PSS is used. The long-term stability of the presented free-floating neural probes is demonstrated in vitro and in vivo. The promising results suggest the feasibility of these neural probes for chronic applications.

## 1. Introduction

Understanding the complex functionality of the brain is the major objective in neuroscience. For this interesting field of research, adequate tools are fundamental to perform in vivo experiments to investigate processing in the neural network of the brain. To allow investigation of the natural behavior of the neurons, in vivo experiments are commonly performed with awake laboratory animals. Ideally, for these experiments, neural interfaces are implanted chronically to have the system in a stable homeostatic condition over the course of the experiment or over prolonged periods of time without short term processes of the immune defense. However, the long-term stability of chronically implanted neural probes is still a challenge in this research field. Especially for medical applications, e.g., neuroprosthetics, the neural interfaces must be stable for several years. Due to the foreign body response of the neural tissue to the implanted probes, a glial scar is formed around the probe, which electrically insulates the microelectrodes [1]. This chronic response leads to a decrease in the signal-to-noise ratio over time [2].

In recent years, many research groups have investigated different approaches to solving this “chronic challenge” [3,4,5]. The essential approach of these developments is the reduction of the probe stiffness using soft biocompatible polymer materials like parylene-C, polyimide, and SU-8 [6]. Using these flexible materials the mechanical coupling between the implanted probe and the skull can be reduced significantly, which leads to a reduced inflammatory response of the neural tissue during brain micromotion [7,8].

Recently, Luan et al. showed stable chronic neural recordings with glial scar-free neural integration of fully-flexible SU-8-based probes [9]. However, the insertion of these fully-flexible probes into the cortex is a challenge. Luan et al. used a microneedle, which is anchored through a microhole at the tip of the flexible probe and afterwards inserted into the cortex [9]. This shuttle device is removed afterwards to leave only the flexible probe inside the cortical tissue. However, the handling of such a device is complicated and a precise insertion into a specific target of the brain is difficult.

Another method to allow the insertion of fully-flexible probes is the temporary increase of the probe stiffness by using biodegradable coatings like polyethylene glycol (PEG), saccharose, maltose, and silk [10,11,12,13]. After insertion of these probes, the coating dissolves into the brain tissue to release a flexible probe. However, due to the additional stiffening coating, the overall dimensions of the probes increases, which leads to an increased lesion in the cortex. Furthermore, the time for insertion of these probes is limited due to the dissolution process of the coatings.

Also, a hybrid integration of a silicon-based probe with a polymer-based flexible ribbon cable is possible to reduce the mechanical coupling between the inserted probe and the skull-fixed connector. Barz et al. presented recently a method for a hybrid integration of a silicon shaft with a polyimide ribbon cable of the same width to allow a complete insertion of the silicon shaft into the cortex, which enables a floating operation [14]. However, the hybrid approach limits the channel density of the probes due to the required pads on both parts for the flip-chip bonding process. Furthermore, an additional biocompatible coating is necessary to encapsulate these interconnections, which also increases also the cross-sectional dimension of the probes.

**Figure 1:**
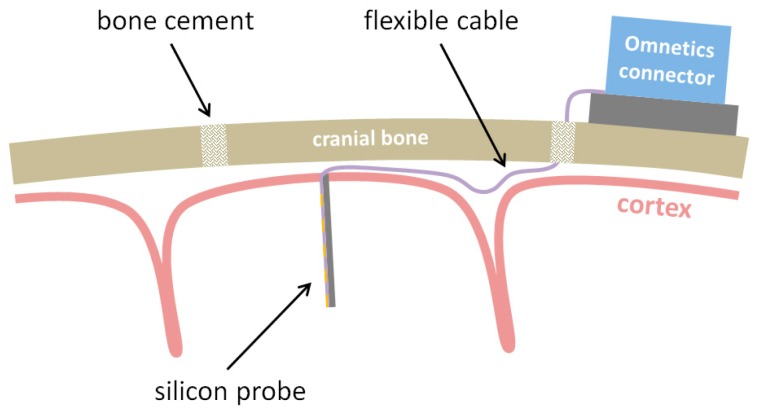
Schematic of a free-floating implantation of an intracortical silicon-stiffened neural probe to reduce the mechanical coupling between the cortex and the skull-fixed connector during brain micromotion [15].

To overcome these limitations, we recently introduced novel flexible neural probes with silicon-stiffened shafts, which can be inserted completely and precisely into the cortex to allow a free-floating implantation (see Figure 1) [15,16]. For this purpose, a microfabrication process was developed, which allows a monolithic integration of a silicon-stiffened neural probe with a highly-flexible polyimide-based cable at a high resolution of 1 µm. Compared to the already reported microfabrication process, this paper presents the latest microfabrication development with the focus on the long-term stability of the used materials polyimide and the conductive polymer PEDOT:PSS, which shows superior properties regarding electrode impedance and charge injection capacity [17,18]. Furthermore, a new silicon-based, vacuum-actuated insertion tool is presented, which allows easy handling of the floating probes during the surgery procedure. The long-term stability of the polyimide electrical insulation is demonstrated in vitro. In vivo recordings of neural activity are shown using chronically implanted probes in rat cortex.

## 2. Materials and Methods

### 2.1. Design of the Floating Neural Probes

**Figure 2:**
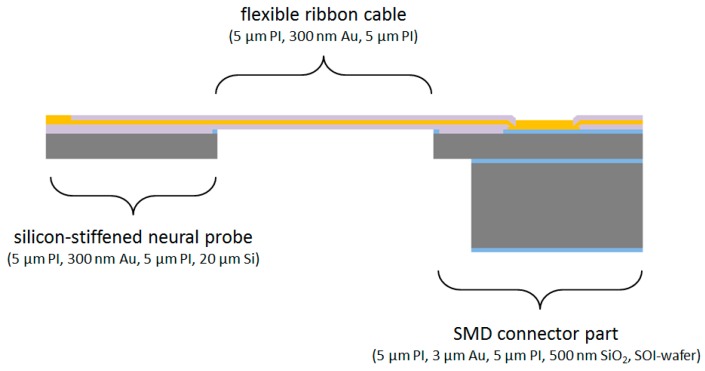
Cross section view of the general neural probe design with three monolithically integrated parts: silicon-stiffened neural probe, highly-flexible ribbon cable, and connector part for soldering of a SMD connector.

The floating neural probes are designed for the chronic implantation into the visual cortex of non-human primates to investigate the causal role of specific neuronal activity patterns and their inter-areal coordination for attention-dependent neuronal signal routing between different cortical areas. For this purpose, the probe shafts have a length between 2 and 5 mm depending on the brain areas of interest. To achieve a sufficient stiffness of the probes for insertion a 20 µm thick silicon layer is used. The biocompatible polymer polyimide with the total sandwich layer thickness of 10 µm is used as the flexible material for the electrical insulation of the microelectrodes and the conductive paths. Compared to parylene-C, polyimide has a significantly higher mechanical stability [6], which is required for this application. Furthermore, the electrical insulation stability in saline using polyimide shows superior results as compared to parylene-C [19]. For the microelectrodes and the conductive paths gold is used as a biocompatible metall. In total, 18 microelectrodes are implemented on the probe shaft, including microelectrodes with a small electrode area of 160 µm^2^ for spatially high-resolution recordings and larger area electrodes of up to 4000 µm^2^ for electrical stimulation. Using a pitch of 5 µm for the 18 conductive paths, the maximum width of the probe shaft is only 130 µm. Due to the monolithic microfabrication process the flexible interconnection to the probe shaft has the same width at the transition point. The flexible cable, up to 60 mm long, ends at a rigid connector part for soldering and encapsulation of an 18-pin SMD Omnetics connector. The three monolithically integrated parts are shown in Figure 2.

### 2.2. Microfabrication Process of the Floating Neural Probes

For the monolithic microfabrication process, silicon-on-insulator (SOI) wafers must be used to define the thickness of the silicon for probe stiffening and to allow a well-defined etching stop during the deep reactive ion etching (DRIE) of silicon. 100 mm SOI wafers with a 20 µm thick device layer, 500 nm thick buried oxide (BOX) layer, and 400 µm thick handling layer are used for this purpose (Silicon Materials, Kaufering, Germany). The total microfabrication process can be divided into two parts: processing of the wafer device layer and processing of the wafer handling layer.

#### 2.2.1. Processing of the Wafer Device Layer

After the wafer cleaning step in Caro’s acid, the wafers are thermally oxidized using a wet oxidation process at 1000 °C to achieve a 500 nm thick SiO_2_ layer. This layer serves as a masking layer for the DRIE processes and as an electrical insulation layer to the silicon substrate at the connector part for the soldering pads. In the next step the soldering pads are realized by DC sputtering of a 100 nm thick titanium adhesion layer and a 500 nm thick gold layer. These layers are afterwards structured using a 1.8 µm thick positive photoresist AZ1518 (MicroChemicals GmbH, Ulm, Germany). Gold and titanium are wet-chemically etched in Au Etch 200 (NB Technologies GmbH, Bremen, Germany) and Buffered Oxide Etch BOE 7:1 (MicroChemicals GmbH, Ulm, Germany), respectively. The photoresist is afterwards stripped using AZ 100 Remover (MicroChemicals GmbH, Ulm, Germany). In the next step, the 500-nm-thick thermal oxide layer is structured using 1.8 µm thick AZ1518 photoresist and a CF_4_/CHF_3_ reactive ion etching process to create the masking layer for the DRIE process and to remove the oxide layer on the probe shaft due to the high compressive stress of SiO_2_, which can lead to an undesired bending of the probe. The photoresist is afterwards stripped in AZ 100 Remover. To guarantee a strong adhesion between silicon/silicon oxide and polyimide an adhesion promoter is used directly before coating with polyimide. An aqueous solution of 0.1 vol.-% of 3-Aminopropyltriethoxysilane (Sigma-Aldrich Chemie GmbH, Munich, Germany) is prepared, spin-coated at 4000 rpm for 20 s, and dried for 2 min at 120 °C using a hotplate. Directly afterwards, polyimide U-Varnish S (UBE Europe GmbH, Düsseldorf, Germany) is spin-coated at 3000 rpm for 60 s and cured using a stepped temperature profile up to 450 °C according to the product manual and a vacuum-hotplate (UniTemp GmbH, Pfaffenhofen an der Ilm, Germany), resulting in a layer thickness of 5 µm. This polymer layer is afterwards structured using a 10 µm thick AZ9260 photoresist (MicroChemicals GmbH, Ulm, Germany) and an O_2_/CF_4_ reactive ion etching process to open the gold soldering pads at the connector part. This is necessary to guarantee a mechanically stable substrate for the soldering process of a SMD connector. The photoresist is afterwards stripped using AZ 100 Remover. In the next step, a 300 nm thick gold layer is deposited using a DC sputtering process. This layer is structured using the 1.8 µm thick AZ1518 photoresist and the Au Etch 200 etching solution to fabricate the microelectrodes, the 2 µm wide conductive paths on the probe shaft and flexible cable and the interconnections to the soldering pads. The photoresist is afterwards removed in AZ 100 Remover. In the next step, the second 5 µm thick polyimide layer is spin-coated and cured as described previously. To achieve a strong adhesion to the first polyimide layer, which is fundamental for the long-term stability of the flexible neural implants, a short oxygen plasma treatment (30 s) using a reactive ion etching process has to be done directly before the coating process. The second polyimide layer is then structured using a 10 µm thick AZ9260 photoresist and an O_2_/CF_4_ reactive ion etching process to open the microelectrodes and the soldering pads. To increase the microelectrode stability and enable soldering of a SMD connector, the thickness of the opened gold layer is additionally increased to approx. 3 µm by gold electroplating using NB Semiplate Au 100 electrolyte (NB Technologies GmbH, Bremen, Germany). Afterwards, the photoresist is stripped using AZ 100 Remover.

**Figure 3:**
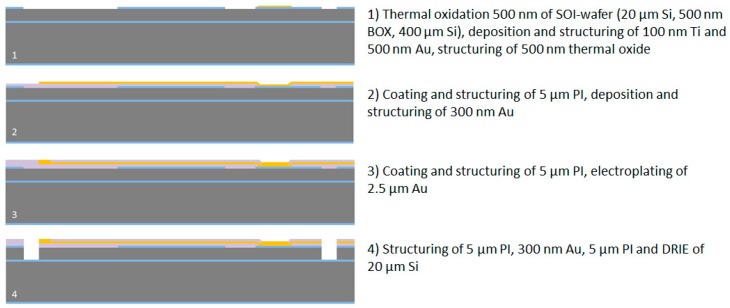
Cross section view of the microfabrication process flow for the wafer device layer.

To define the shape of the neural probe, the flexible cable and the connector part, the polyimide/gold/polyimide sandwich structure is patterned in the following steps. First, a 10 µm thick AZ9260 photoresist and a O_2_/CF_4_ reactive ion etching process is used to etch the second polyimide layer and afterwards the electrical connections to the soldering pads for the electroplating process by wet-chemical etching in 0.5 mol/L iodine solution (Grüssing GmbH, Filsum, Germany). After removing the photoresist in AZ 100 Remover, a second 10 µm thick AZ9260 photoresist is used to pattern the first polyimide layer using an O_2_/CF_4_ reactive ion etching process. Afterwards, the 20 µm thick silicon layer is structured using a DRIE process with a combination of the photoresist and the thermal oxide layer as the etching mask. The etching process stops well-defined on the buried oxide layer of the SOI wafer. Before stripping the photoresist in AZ 100 Remover, a short oxygen plasma treatment (30 s) is applied to remove the passivation layer produced during the DRIE process. The processing of the wafer device layer is herewith completed, see Figure 3.

#### 2.2.2. Processing of the Wafer Handling Layer

To enable the release of the neural probe, the integrated flexible cable and the connector part after fabrication from the wafer, a multi-step DRIE process is used. Before continuing structuring of the wafer handling layer, the wafer device layer is protected against scratches using a 1.8 µm thick AZ1518 photoresist. Furthermore, this layer serves at the end as a mechanical anchor of the probes before releasing them from the wafer.

For the multi-step DRIE process, two successive masking layers are used, namely silicon oxide and photoresist. In the first step the 500 nm thick thermal silicon oxide is structured using the 1.8 µm thick AZ1518 photoresist and the CF_4_/CHF_3_ reactive ion etching process to open the oxide mask underneath the flexible ribbon cable. After removing the photoresist in AZ 100 Remover, a 10 µm thick AZ 9260 photoresist is used for the following DRIE processes. Note that, after stripping the photoresist, the protective coating with the 1.8 µm thick AZ1518 photoresist has to be applied again on the wafer device layer. In the first DRIE process step, approx. 80 µm silicon is etched underneath the flexible ribbon cable using silicon oxide as the etching mask. Afterwards, the silicon oxide layer is removed selectively by using a CF_4_/CHF_3_ reactive ion etching process. In the next step, the DRIE process is continued until the buried oxide of the SOI wafer is opened completely underneath the flexible ribbon cable. This buried oxide layer is afterwards etched using a CF_4_ reactive ion etching process.

**Figure 4:**
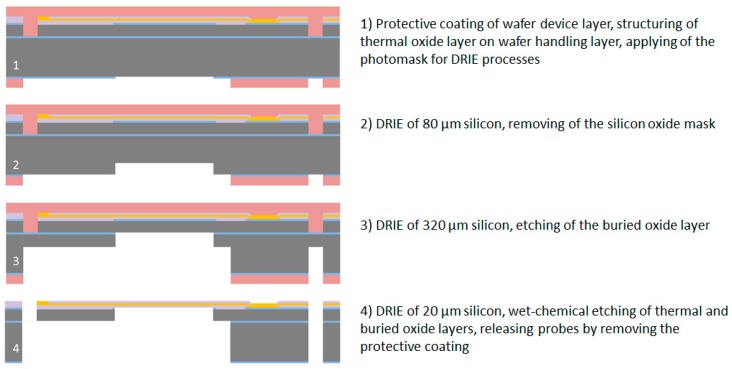
Cross section view of the microfabrication process flow for the wafer handling layer.

In the final DRIE process step, the silicon underneath the ribbon cable is removed completely and at the same time the buried oxide underneath the probe shaft is opened. To remove the remaining thermal and buried oxide, a wet-chemical etching process with Buffered Oxide Etch (BOE) 7:1 is used. The neural probes and connector parts are now fixed to the silicon wafer only by the protective coating (1.8 µm photoresist) on the wafer device layer. Finally, this protective coating is removed in AZ 100 Remover to release the neural probes from the wafer. The processing of the wafer is herewith completed, see Figure 4.

### 2.3. Soldering and Encapsulation of an Omnetics SMD Connector

After the microfabrication process, an 18-pin SMD Omnetics connector (NPD-18-VV, Omnetics Connector Corporation, Minneapolis, MI, USA) with a fine pitch of 0.64 mm is soldered on the connector part of the neural probe. For the reflow soldering process the soldering paste LFM-65W A75C(L) 12% (Almit GmbH, Michelstadt, Germany) is used, which has an eutectic temperature of 139 °C. The precise alignment of the connector and the soldering process is done with a FINEPLACER tool (Finetech GmbH & Co. KG, Berlin, Germany). After the soldering process, the connector part of the neural probe is additionally encapsulated using the two-component epoxy adhesive UHU PLUS 300 (UHU GmbH & Co. KG, Bühl, Germany). To increase the mechanical stability the adhesive is also cured at 100 °C for 20 min. As the connector will be fixed on top of the skull without a contact to biological tissue, an additional biocompatible coating of the connector part is not necessary.

### 2.4. Microelectrode Coating with PEDOT:PSS

To reduce the impedance of the gold microelectrodes and enable electrical stimulation, the electrodes are finally coated with the conductive polymer poly(3,4-ethylenedioxythiophene) polystyrene sulfonate (PEDOT:PSS). For this purpose, an electropolymerization process is used, which allows a long-term stable coating proved by previous in vitro experiments [20]. The monomer aqueous solution consists of 2 wt% PSS (Mw ~70,000, Sigma-Aldrich Chemie GmbH, Munich, Germany) and 10 mmol/L EDOT (Sigma-Aldrich Chemie GmbH, Munich, Germany). The mixture is prepared using a magnetic stirrer and an ultrasonic bath. For the polymerization process, a platinum counter electrode and a precise constant current source (CompactStat, Ivium Technologies, Eindhoven, The Netherlands) are used. All microelectrodes on the probe shaft are coated simultaneously at a current density of 5 µA/mm^2^ and a deposition time of 400 s. For a homogenous and reproducible coating, the monomer solution is additionally stirred slowly during the electropolymerization process using a magnetic stirrer at approx. 50 rpm. To significantly increase the adhesion strength between the PEDOT:PSS layer and the gold layer, the surface of the electroplated gold microelectrodes is partially wet-chemically etched directly prior to the PEDOT:PSS coating [21]. Using a diluted 0.05 mol/L iodine solution (Grüssing GmbH, Filsum, Germany) and an etching time of 1 min a porous gold surface is created, which allows a mechanically strong bonding of the PEDOT:PSS coating to the gold substrate.

### 2.5. Microfabrication of the Vacuum-Actuated Insertion Tool

**Figure 5:**
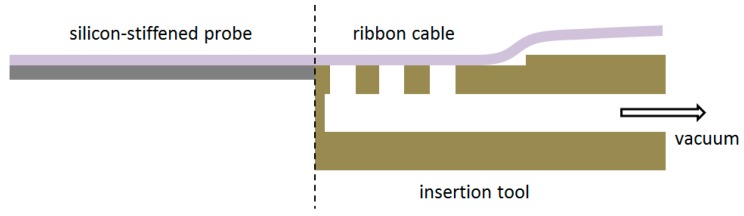
Schematic diagram showing the principle fixation of the floating probe using the vacuum-actuated insertion tool.

To allow the implantation of free-floating neural probes, the silicon-stiffened shaft must be inserted completely into the cortical tissue, as shown in Figure 1. Previously, we developed a silicon-based insertion tool for this purpose, where the neural probe is fixed temporarily with a drop of polyethylene glycol (PEG) [15]. However, this approach has two drawbacks, namely the limited time during insertion due to the dissolving process of the PEG with contact to the brain liquid and the complicated alignment of the flexible probe into the etched channel and the following fixation with hot PEG. To overcome these limitations, a silicon-based microfabrication process for an insertion tool with an integrated microfluidic channel is developed to achieve the fixation of the probe with a vacuum exhaust, see Figure 5.

**Figure 6:**
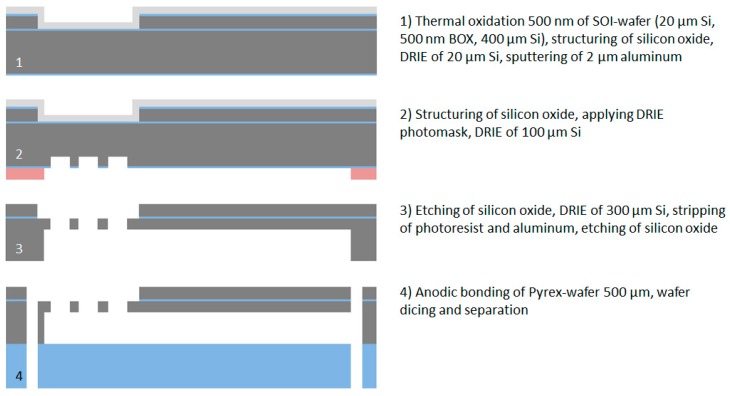
Cross section view of the microfabrication process flow for the vacuum-actuated insertion tool.

For the microfabrication of the vacuum-actuated insertion tool, the same SOI wafer is used as for the neural probe fabrication. After the thermal oxidation (500 nm) of the wafer, the silicon oxide of the device layer is structured by a CF_4_/CHF_3_ RIE process followed by DRIE of 20 µm silicon to create the channel for the alignment of the flexible ribbon cable. To achieve a mechanically stable etching stop layer for the DRIE process, a 2 µm thick aluminum layer is DC sputtered on the device layer. The buried microfluidic channel and the vacuum inlets for the fixation of the probe are produced by a multi-step DRIE process similar to the microfabrication process of the neural probes. After structuring of the silicon oxide of the handling layer by a CF_4_/CHF_3_ RIE process, a 10 µm thick AZ9260 photoresist is applied and the first DRIE step is performed to etch ca. 100 µm silicon. After removing of the silicon oxide using a CF_4_ RIE process, the DRIE process is continued until the buried oxide of the SOI wafer is completely opened. In the next step, the photoresist is removed in AZ 100 Remover. The aluminum layer is selectively stripped in phosphoric acid etching mixture PWS 80-16-4 (Honeywell Specialty Chemicals GmbH, Seelze, Germany) and the oxide layers are removed using Buffered Oxide Etch BOE 7:1. Directly prior to the anodic bonding process, the SOI wafer and the Pyrex wafer are cleaned using Caro’s acid solution. The anodic bonding is performed at a temperature of 400 °C and a peak voltage of 600 V. Finally the bonded wafer is diced to separate the individual insertion tools with the overall dimension of 1 × 0.9 × 40 mm (width × thickness × length). The total microfabrication process flow is shown in Figure 6.

### 2.6. In Vitro Evaluation of the Electrical Insulation Stability of Polyimide

The chronic application of floating neural probes requires a long-term stability of the used polyimide U-Varnish S (UBE Europe GmbH, Düsseldorf, Germany) electrical insulation of the conductive paths at the flexible cable and the probe shaft. Therefore, an in vitro long-term test is performed using Ringer’s electrolyte solution to evaluate the diffusion of the electrolyte into the polymer layers over time. For this purpose, two microfabricated neural probes are used with 18 gold microelectrodes (160 µm^2^) each. The probe shaft and approx. 40 mm of the flexible cable are immersed into the Ringer’s solution inside a sealed glass container. The electrolyte is additionally tempered to a constant body temperature of 37 °C using a hotplate and a temperature sensor to imitate in vivo conditions. The insulation stability is monitored using impedance measurements (V_pp_ = 100 mV, CompactStat, Ivium Technologies, Eindhoven, The Netherlands) at a frequency of 1 kHz between adjacent conductive paths. To increase the measurement sensitivity of the parasitic capacitance formed by the dielectric polyimide layer in between the conductive paths, the gold microelectrodes are not coated with the polymer PEDOT:PSS to achieve a high initial impedance. For chronic implantation, the probes must be sterilized. To include any physical and chemical changes of the polyimide layers by the used steam sterilization process (121 °C, 1 bar, 20.5 min) the samples are additionally steam sterilized before long-term stability evaluation.

### 2.7. Implantation Procedure of Floating Neural Probes and Electrophysiological Recording

All procedures used in this study were performed in accordance with the guidelines for the welfare of experimental animals issued by the Federal Government of Germany, approved by local authorities and conformed to the guidelines of the National Institute of Health for the care and use of laboratory animals.

The vacuum-actuated insertion tool was glued into a 14 g medical cannula with a two-component instant adhesive (Loctite 3090, Henkel AG & Co KgaA, Düsseldorf, Germany) in order to be able to connect it to a medical suction device (Medutek Kataspir30, Medutek, Bremen, Germany) via an infusion line. This assembly may be steam sterilized and can be attached to any commercially available electrode drive. In our case, we used a precision micro-manipulator by TSE (TSE-Systems, Bad Homburg vor der Höhe, Germany). The sterilized neural probe is assembled to the insertion tool while generating a vacuum of −0.8 bar max. The exact position of the neural probe inside the groove of the insertion tool is adjusted with plastic-tipped precision forceps. This process takes about one minute.

The silicon-based probes were implanted chronically into the primary visual cortex of rats. The rats were anesthetized with an intraperitoneal injection of ketamine/medetomidine (6 mg/50 µg/100 g body weight). During surgery, the animal was placed in a stereotactic system. The scalp was removed and a small (2 × 2 mm) bone window above the primary visual cortex was drilled (centered at AP = +2 mm and L = 2.5 mm from Lambda). Before insertion of the neural probe the dura was reflected. After insertion, the ribbon cable is left free-floating, the bone window is reinserted into the craniotomy and the cuts are filled with resorbable gel-foam (Gelastypt, Sanofi, Frankfurt am Main, Germany). Finally, the craniotomy is covered with dental acrylic. The Omnetics connector is anchored to the skull with 4–5 bone screws and dental acrylic.

During recordings of neural activity the animals were anesthetized with 2% isoflurane. Electrophysiological data was recorded with an open-source recording system [22], based on Intan amplifier chips (Intan Technologies, Los Angeles, CA, USA) without any visual stimulation. The electrode signals were referenced to ground, filtered between 0.1 and 6000 Hz, multiplexed, and digitized at 30 kHz on the amplifier chip.

## 3. Results and Discussion

### 3.1. Microfabrication of Free-Floating Neural Probes

**Figure 7:**
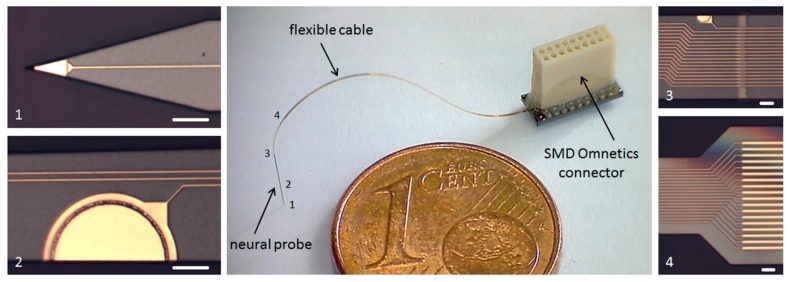
Microfabricated silicon-stiffened neural probe (5 mm long, 30 µm thick and max. 130 µm wide) with monolithically integrated 25 mm long flexible cable (10 µm thick PI) connected to a soldered and encapsulated 0.64 mm pitch 18-pin SMD Omnetics connector with enlarged top view of (**1**) probe tip with a 160 µm^2^ microelectrode (scale bar 30 µm); (**2**) probe shaft with a 3000 µm^2^ microelectrode (scale bar 30 µm); (**3**) transition of the probe shaft with a 160 µm^2^ microelectrode to the flexible ribbon cable (scale bar 20 µm); (**4**) widening of the gold conductive paths 3 mm after the probe/cable transition from 5 µm to 10 µm pitch (cable width increases from 130 µm to 230 µm, scale bar 15 µm).

Using the presented microfabrication process flow 18-channel, free-floating neural probes with monolithically integrated highly-flexible ribbon cables were successfully produced. Different probe designs with probe lengths between 2 and 5 mm and cable lengths between 25 and 60 mm are realized to match the final in vivo application. For the electrical interface, a fine-pitch Omnetics connector is soldered and electrically encapsulated (see Figure 7).

Compared to the previous published process [15], the current microfabrication yield of these probes could be improved to approx. 70%. The main defects during the fabrication process occur due to particles in the photoresist layers. The total microfabrication process is complex, but reproducible. Especially the multi-step DRIE process flow for releasing the probes from the SOI wafer is sensitive to the current condition of the used etching tools; therefore, a sufficient conditioning is important to guarantee reproducible etching results. The vacuum-cured polyimide layers show no bending of the ribbon cable and the probe shaft after fabrication, thus the layer stress of the two polyimide layers is negligible. Compared to previous reported parylene-C-based flexible ribbon cables [23] the mechanical stability of the narrow polyimide-based cables is significantly higher, which is essential for a reliable handling of the probes during the surgery.

### 3.2. Coating of the Gold Microelectrodes with PEDOT:PSS

**Figure 8:**
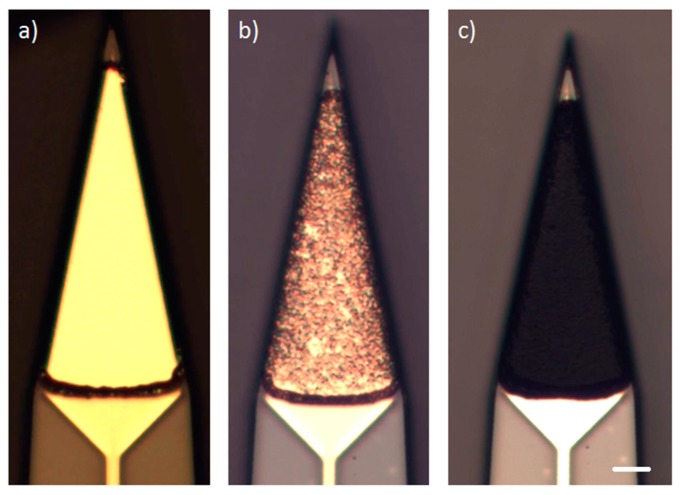
Microscopic top view of the microelectrodes (3000 µm^2^) at the probe tip showing (**a**) smooth electroplated gold surface after microfabrication; (**b**) rough gold surface after etching in 0.05 mol/L iodine solution for 60 s; (**c**) PEDOT:PSS coated microelectrode (scale bar 10 µm).

To increase the adhesion strength between the PEDOT:PSS layer and the electroplated gold surface, the microelectrodes were selectively etched in 0.05 mol/L iodine solution prior to the electropolymerization process of PEDOT:PSS [21]. Figure 8 shows the microelectrode surface before the etching step, after the etching step and after the PEDOT:PSS coating process.

By using this process flow the in vitro impedance at 1 kHz of 160 µm^2^ gold microelectrodes can be significantly reduced from approx. 3 MΩ (electroplated gold surface) to 133 ± 11 kΩ (PEDOT:PSS coating). Furthermore this coating enables electrical stimulation due to the increased electrode double-layer capacity. In vitro experiments revealed a charge injection capacity of approx. 2 mC/cm^2^ for this PEDOT:PSS coating [16]. This value is comparable to reported in vitro results of PEDOT:PSS coated microelectrodes by Venkatraman et al. [24]. Also, the pretreatment process for increasing the adhesion strength of PEDOT:PSS on a gold surface is simple compared to the application of additional adhesion layers e.g., nanostructured platinum [25].

### 3.3. Microfabrication of the Vacuum-Actuated Insertion Tool

**Figure 9:**
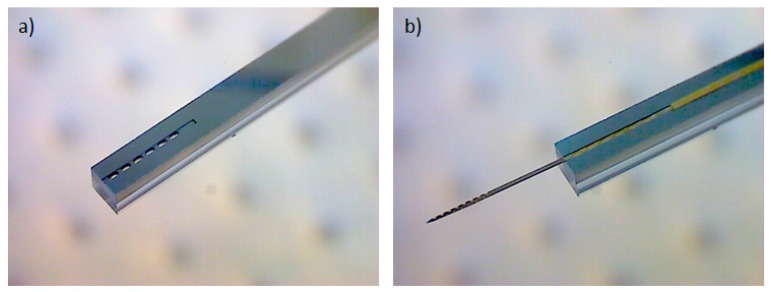
Side view of the silicon-based vacuum-actuated insertion tool (1 mm wide, 0.9 mm thick, and 40 mm long) with an integrated microfluidic channel (**a**) and a vacuum-fixed floating neural probe for complete and precise insertion of the silicon-stiffened shaft into the cortex (**b**).

Using the described microfabrication process flow vacuum-actuated, biocompatible insertion tools were successfully produced. A 20 µm deep, 150 µm wide, and 3 mm long trench on the top side of the insertion tool allows easily a precise alignment of the flexible ribbon cable parallel to the insertion tool. The ribbon cable is fixed by the openings of the integrated vacuum channel, see Figure 9.

Due to the 20 µm thick silicon layer underneath the polyimide sandwich layers of the probe shaft, a mechanically stable joint is created at the probe shaft and insertion tool transition to allow a successful insertion of the probe into the cortex. The presented insertion tool can be easily attached to commercially available micromanipulator devices for surgery. The vacuum can be realized using a silicone tube connected to the opening of the integrated channel at the other end of the insertion tool and a vacuum pump. Mechanical tests showed a sufficient stable fixation of the floating neural probes until the maximum buckling force of 10.8 ± 0.3 mN for 5 mm long probes. Using this new tool, it is now possible to implant also several floating probes into the cortex within a short period of time, which is a main advantage over the application of bioresorbable coatings, e.g., saccharose [11].

### 3.4. In Vitro Evaluation of the Polyimide Long-Term Electrical Insulation Stability

**Figure 10:**
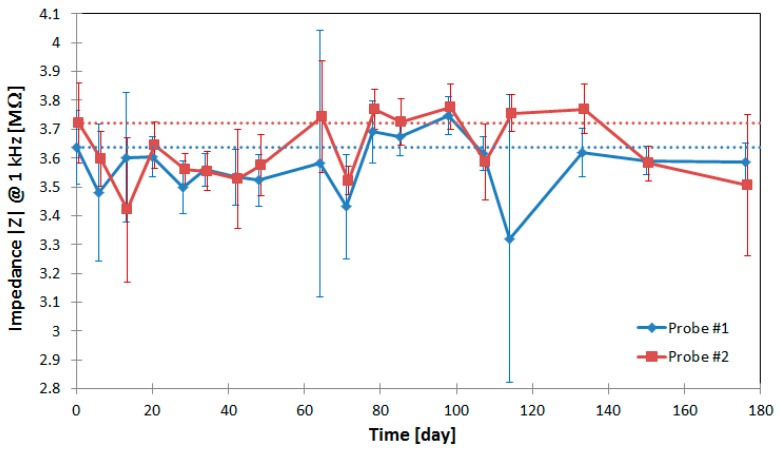
In vitro impedance long-term measurement results between adjacent conductive paths on the flexible cable and the probe shaft for two floating neural probes show an impedance reduction of 1.4% for probe 1 and 5.8% for probe 2 after almost six months.

The long-term electrical insulation stability of the polyimide layers is fundamental for chronic applications [6]. For this purpose, two neural probes are immersed in Ringer’s solution at 37 °C and the impedance between the adjacent 18 conductive paths is measured over time to investigate the influence of the diffusion of saline into the polymer layers on the dielectric properties. As the measured parasitic capacity is parallel to the serial impedance of two microelectrodes, the electrodes are not coated with PEDOT:PSS to allow a higher measurement sensitivity of the electrolyte diffusion. The results of the long-term evaluation are shown in Figure 10. The impedance at 1 kHz after almost six months was reduced 1.4% for probe 1 and 5.8% for probe 2. These results confirm the increase of the parasitic capacitance due to the diffusion of saline into the polymer layers. However, this increment is negligible compared to the low impedance of PEDOT:PSS coated microelectrodes. Therefore, only a minimal increase of the signal cross talk is possible.

### 3.5. In Vivo Demonstration of Chronic Intracortical Recording

**Figure 11:**
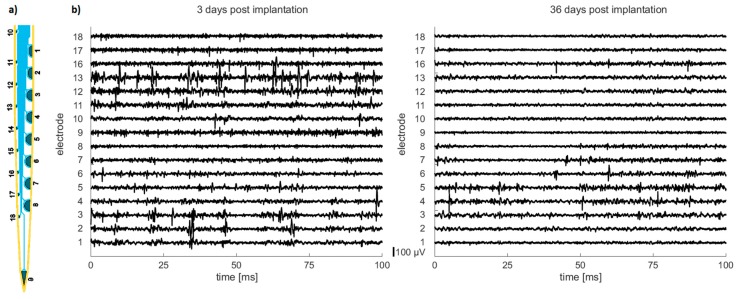
In vivo chronic recordings of spontaneous neural activity in rat visual cortex using a 2 mm long silicon-based free-floating probe with 18 microelectrodes (**a**) 3 days and 36 days after implantation demonstrate the long-term capability of recording single unit activity (**b**).

To evaluate the long-term functionality of the free-floating probes, a fabricated 2 mm long neural probe with 18 PEDOT-coated microelectrodes (see Figure 11a) was implanted chronically into the visual cortex of a rat with the help of the new vacuum-actuated insertion tool.

Figure 11b shows 100 ms stretches of bandpass filtered data (0.3–5 kHz) obtained 3 days (left graph) and 36 days (right graph) after implantation. Due to technical constraints, we were able to record from 16 out of the 18 electrodes only. The data was collected during an experiment investigating the neural representation of visual stimuli in neurons across all six layers of the primary visual cortex under anesthesia. For comparability reasons, the data presented here are taken during periods without a visual stimulus presented to the system, i.e., the neurons under investigation are displaying spontaneous activity only. During visual stimulation, the activity of a large fraction of neurons is strongly enhanced depending on their individual stimulus preference.

In both recordings, single unit activity can be identified in small-area (160 µm^2^) and large-area (3000 µm^2^) microelectrodes characterized by the temporally sharply defined voltage transients of approximately 1 ms duration visible in the recorded data. In addition, there is little evidence for cross talk between adjacent conductive paths for both days. The average signal-to-noise ratio (SNR) of 16 recorded channels 3 days after implantation is 24.6 ± 9.9 and 36 days after implantation 22.8 ± 7.7. This corresponds to a SNR reduction of ca. 7%, which is acceptable for chronic applications.

## 4. Conclusions

The presented silicon-based microfabrication process allows the monolithic integration of a multichannel neural probe with a narrow and highly flexible ribbon cable for a free-floating implantation in the cortex. A high resolution of 1 µm can be achieved also at the probe to cable transition, which reduces the mechanical impact of the flexible interconnection. The improved adhesion strength between two polyimide layers by an O_2_ RIE process guarantees the long-term stability of the electrical insulation, which is essential for chronic applications and proved by an in vitro long-term evaluation. Also the adhesion strength between a PEDOT:PSS and a gold layer is significantly increased by an iodine etching process directly before the electropolymerization of the monomer EDOT.

A new vacuum-actuated insertion tool is presented, which allows for a fast, complete, and precise insertion of the neural probes and simplified handling during the surgery. In vivo recordings of neural activity in rat cortex with a chronically implanted probe demonstrate the long-term functionality. These results are promising, e.g., for neural prosthetics.

Further in vivo experiments have to be done to evaluate the free-floating approach and a direct comparison with skull-fixed probes can help to understand the chronic tissue response.
